# Present Status of Sex Education

**Published:** 1914-12

**Authors:** G. Henri Bogart

**Affiliations:** Paris, Illinois


### The Department of Eugenics, Mental and Social Hygiene

CONDUCTED BY
DR. MALONE DUGGAN
813-814 Gibbs Building, San Antonio, Texas
All communications for this department should be sent to DR. DUGGAN

### Present Status of Sex Education.

BY DR. G. HENRI BOGART, PARIS, ILLINOIS.

The wave of public furore concerning sex education has undergone a revulsion, the tide has swept back, and progress appears to have been lost, though such is far from truth.

All progress has been in cycles, alternating periods of advance and retreat.

Action and reaction are equal in contrary directions, and the pendulum of destiny swings ever between the extremes of its arc, with no apparent advancement, but, hidden up above, the wheels turn ever so slowly, and the hands creep round the dial, until in some glad day they shall stand, pointing together toward the zenith, and it shall strike high noon for humanity.

When first the subject of eugenics was given to the public it ran round the earth like wildfire in dry grass, and the world, which like the Athenians on Mars Hill, is still seeking the "New Thing," devoured the theme greedily and without discrimination.

The vice commissions, the white slave agitation and the sterilization of the unfit were kindred questions to the laity, in that they all dealt with the organs of reproduction, and all these with any correlative incidents were tortured and twisted and distorted into dishes labeled "eugenics" and fed to the popular appetite.

Indirectly, all of these questions do have to do with the teachings of eugenics, which means the bringing into the world of well-born children.

Cupidity found a new field of exploitation, and the masters of the yellow journals sent their young men out into the field, commanding that the popular appetite be fed with something new, well spiced, and the man whose trade is that of exaggeration and sensationalism had to bring in some sort of a story to hold his job, and the nearer the story approached the limits of the allowable, or the wilder its spice of improbability or novelty, the better it suited the editors.

It must be remembered, too, that those reporters had no special knowledge to enable them to sift the ludicrous and the impossible from the truth.

Two other classes saw a market in the popular interest—the mercenary and the fanatic. So the platform and the printing press alike swarmed with those who knew little about the subject, the one class intent on winning coin, the other honestly attempting to teach their half-baked ideas, and in this the more dangerous with that little learning which is a dangerous thing.

The market, too, was a peculiarly receptive one, for from that day, when the Phallus was worshipped as the physical manifestation of the creative power, down to today, there is a curiosity, proper and laudable, as to the reproductive functions and organs, which ever demanded answer.

So with the onslaught on the citadels of prudery, and the sudden opening of that question which the whole of humanity has ever held as of prime importance, the forces of intelligence, of ignorance, of cupidity, and of fanaticism joined to make the word eugenics fit everything, from the real science to the veriest drool of pruriency.

Then, the revulsion came as was inexorable, the populace wearied with the reaction, there was a halt, the courts responded to the referendum of the voice of the majority, and some of the laws passed were set aside, while others were relegated to the position of ciphers.

Even this was not unmixed evil, since weak statutes were wiped away; as, for instance, the sterilization farcial regulations of California and New Jersey, leaving a clear field for some future action.

With all this, the seed that was planted had taken root.

A breach has been made into the dark wall of prudery, and there is a body of thinkers in every community; thinkers who know the need and the possibility of a wider and a purer knowledge of reproduction, and even the many whose sole interest was in the nastiness made from the subject got some glimmer of the fact that there is a clean science connected with the coming of a new generation into the world.

The penduium will swing back, there will be a higher plane for the next return to the intelligent study of this vital subject, and the passage of better laws for the control of vice and vicious sexuality will be the easier, for, while the way has not been paved, the road has been plowed, and the pathway, while now more difficult for travel, is partially ready for better progress, when the work is taken up anew.

In the meantime the real leaders of the movement are not cast down with despondency. They recognize the conditions, and they are busily gathering data for the next onslaught against that ignorance which has been held as equivalent to innocency, when the pendulum swings back in its certain cyclic course.

The husbandman does not sink into despair when winter comes, for he knows that spring shall smile, some time, and that the temporary lull in fruition is a part of nature's course.

The conflict of Europe will have a direct influence on the sexual status. Men, the most virile of the population, are being slaughtered by the thousands, children will die from privation, and, already, the lay press tells the story of plans being made for the increase of European birth rate, even while the conduct of the campaigns is engaging the attention of the governments.

It is well to remember that much of our sexual convention is rather the result of usage than of intrinsic right and wrong.

Polygamy and polyandria, concubinage, trial marriage and other phases of sexual relationships which appeal to the average citizen as horribly gross and wicked have been the ideals of the highest intelligence of the world at certain periods, and our standards would have been held as equally wrong in those civilizations. The urgency of need for increased population sets the standards of sexual morality.

Do not understand that I am defending those customs; I am but calling attention to the differences of different conventions.

The sexes exist that the race may reproduce itself, sexuality is part of the Divine plan, and the various standards of human limitation to the application of reproduction are but human after all.

Eugenics, the application of common sense and science to the breeding of the race, is as certain to elevate the standard of humanity as it has of the fruits and the domestic animals, though, as a matter of course, there will ever remain the elements of love and personal selection.

At the same time, we should consider some of the basic wrongs of our conventionality. The woman who sells her person, in wedlock, for the consideration of wealth and position, is held to have achieved the summit of marital success, while her sister who sells her charms in the oldest profession is placed at the other end of the scale. This shows what I mean by the conventionality of our standards of right and wrong in the mating of man and woman. And so, too, of our ideas of divorce on the one hand, and of holding marriage as an indissoluble tie on the other.

Certain it is that we are all agreed that the propagation of the unfit adds to the burden of humanity and lowers the average of the race, though there are differences as to whether this shall be prevented by segregation, by restriction or prohibition of marriage and cohabitation. Certain it is that the individuals who realize their responsibilities and the methods of control will better aid in good births and in limiting bad births. Certain it is that the world needs that the best blood and breeds shall reproduce, though we vary as to the plans and extent of the plans for control and education.

And as the nations of Europe have spent an arduous century in preparation for the carnival of bloodshed, during which all ethical questions of right and wrong have been measured by the ultimate success of the object to be attained, so we who are students, soldiers in the other inevitable conflict for the uplift of the race, should not weary, not be disappointed by the backward swing of the pendulum; rather, we should make ready for the forthcoming reaction, when eugenics, with its fateful importance again swings to the front, that we may be marshalled, prepared to exert the fullest influence to aid in the triumph of the law for the survival of the fittest.

